# Towards universal access to healthcare for older adults: an assessment of the old-age exemption policy under Ghana’s National Health Insurance Scheme

**DOI:** 10.1186/s12939-020-1156-2

**Published:** 2020-03-17

**Authors:** Fidelia A. A. Dake, Nele van der Wielen

**Affiliations:** 1 Regional Institute for Population Studies, University of Ghana, P. O. Box LG 96, Legon, Accra Ghana; 2 Centre for Research on Ageing, University of Southampton, Southampton, UK

**Keywords:** Older adults, Exemption, National Health Insurance Scheme, Ghana

## Abstract

**Background:**

Despite calls for governments to provide universal health coverage for all, social health insurance programmes (SHI) that specifically target older adults continue to be largely absent in many African countries. Only a few African countries have implemented SHI programmes that include specific provisions for older adults. Ghana’s National Health Insurance Scheme (NHIS) is one of the few programmes in Africa that exempts older adults from paying premiums for health insurance. This study examined socio-demographic factors associated with old-age premium exemption under Ghana’s NHIS.

**Methods:**

The study used data from the seventh round of the Ghana Living Standards Survey (GLSS 7) conducted in 2017. Descriptive statistics and binary logistic regression were used in analysing data from a sample of 1532 older adults aged 70 years and older.

**Results:**

The results reveal that only about 43% of older adults who were enrolled on the NHIS at the time of the survey acquired their membership through the old-age exemption policy. Additionally, increasing age was associated with higher odds of reporting exemption from paying premiums for health insurance. Also, older adults who are living in rural areas were more likely to pay premiums rather than being exempt as compared to their counterparts living in urban areas.

**Conclusions:**

These findings indicate that the old-age exemption policy is not achieving the intended goal of providing financial risk protection for some older adults. Additionally, the policy is not reaching those who need it most, particularly those living in rural areas. Specific targeting is required for older adults living in rural areas who are less likely to benefit from the old-age exemption policy in spite of being eligible.

## Introduction

Population ageing is occurring in all regions of the world, albeit to varying extents. It has been noted that the rate of population ageing in low-and-middle income countries is occurring at a faster pace than in high-income countries [1]. Though the African region has the youngest population, the proportion of older adults is increasing. It is estimated that the percentage of the population aged 60 years and older in sub-Saharan Africa will increase from 4.8% in 2017 to 7.6% in 2050 [1]. The growing population of older adults in the African sub-region has several implications that requires specific targeted interventions including health system planning and social health protection specifically for older adults. Such planning is necessary because older adults, particularly those in their later ages, face a greater health burden than other population sub-groups [2]. The rising prevalence of non-communicable diseases and the rise of older adults suffering from multiple conditions (multi-morbidity) [3, 4], puts great pressure on healthcare systems not only in low but also in high income countries. However, while on the one hand older adults have the greatest need for healthcare, on the other hand, they face financial barriers in accessing healthcare because they are among the poorest and most vulnerable population sub-groups who cannot afford to pay for healthcare services [5–7]. Only a minority of older adults in sub-Saharan Africa receive an old age pension, and older adults in low-and-middle income countries spend more per-capita on healthcare than other population sub-groups [7]. The lack of social protection systems for older adults in Africa including in the area of healthcare thus requires urgent attention.

In the African region, only a handful of countries, including Ghana have implemented social health protection programmes that target older adults [7]. The Ghanaian National Health Insurance Scheme (NHIS), which has been operational since 2005, applies an old-age premium exemption policy for older adults to ensure that older adults can access healthcare. The scheme was introduced to improve access to healthcare for all citizens. It provides a benefits package that covers almost 95% of the disease burden in Ghana and includes in-patient hospital care, out-patient care at primary and secondary levels, and emergency and transfer services [8–10]. The NHIS benefit package excludes organ transplant, cancer treatment other than breast and cervical cancer, appliances and prosthesis such as hearing or optical aid [11]. Although the NHIS benefit package does not have a specific focus on geriatric or chronic conditions, the wide range of benefits means that those who are enrolled on the scheme can access basic healthcare. In order to become a member of the NHIS, individuals need to register at the local district office and pay a registration fee as well as a premium. The NHIS premium is based proportionally on people’s income with the poorest and older adults aged 70 and older being exempt from paying any premium fees. The official minimum payment amounts to GH¢7.20 per annum and the upper limit is not allowed to exceed GH¢48.00 [12].

The old-age premium exemption policy applies to older adults aged 70 years and older who are not contributors to the Social Security and National Insurance Trust (SSNIT) pension scheme [13, 14]. Older adults who have contributed to SSNIT can register on the NHIS without paying premiums. It must, however, be noted that SSNIT contributors among pensioners in Ghana are in the minority. Only 0.2% of the percentage of NHIS subscribers are SSNIT pensioners [15]. The old-age premium exemption is seen as an incentive for people aged 70 and above to enrol on the NHIS free of charge to ensure financial risk protection against catastrophic out-of-pocket payments in case of illness.

In order to benefit from the premium exemption, older adults must provide a proof of age [16]. The NHIS guidelines further state that individuals are required to register once in their lifetime but they need to renew their membership annually to be entitled to healthcare coverage under the NHIS [15]. Thus, although the provisions of the old-age premium exemption imply that older adults aged 70 and above do not have to pay a premium when registering under the NHIS, they still need to pay for the annual membership renewal and card processing registration fee.

The old-age exemption policy has been in operation since the implementation of the NHIS but there has been no assessment of the policy. It is, therefore, imperative that the old-age premium exemption policy is assessed to determine whether the intended beneficiaries are being reached and whether the policy is achieving the aim of financial risk protection for older adults. Such an assessment is of utmost importance because Parmar et al. [7] argue that “evidence on whether [social health programmes] have been successful in providing equitable healthcare to older adults where they have access to healthcare on the basis of need, irrespective of their income, age, residency, or sociocultural factors is limited” (p.37). Furthermore, while there has been extensive research on issues of equity and social exclusion with regards to the NHIS [2, 7, 13, 16, 17], there has been very limited research on how these issues affect older adults’ enrolment and access to the scheme. Van der Wielen et al. [18] hold that “despite the premium exemption, richer older adults are still more likely to be part of the NHIS than poorer elders” (p.7–8). They argue that more research is needed to understand whether or not the old-age premium exemption policy is reaching intended targets and achieving the intended benefit of providing financial risk protection from catastrophic out-of-pocket payment for older adults.

This study therefore examines the old age premium exemption policy for older adults under Ghana’s NHIS. To the authors’ knowledge, this is the first study using quantitative methods to understand what enables and what hinders older adults in seeking to benefit from the NHIS premium exception. Specifically, the study seeks to investigate which sub-groups of older adults benefit from the policy and which groups tend to be excluded. We assess the factors associated with exemption or exclusion.

## Materials and methods

### Source of data

This study used data from the seventh round of the Ghana Living Standards Survey (GLSS 7) conducted between 24th October, 2016 and 17th October, 2017 [19]. The GLSS is the Ghanaian version of the Living Standards Measurement Study (LSMS) initiated by the Policy Research Division of the World Bank in 1980 [20]. Ghana has conducted seven rounds of the GLSS since 1987. Data for the GLSS 7 survey were collected using different sets of instruments including a household questionnaire, a community questionnaire, and a questionnaire on prices of food and non-food items [19]. The household questionnaire included different modules (A, B, C and D) with different sections. The sections of Module A of the household questionnaire included demographic characteristics, education and skills training, health and fertility behaviour and migration among others. The section on health contained specific questions on health insurance. The questions on health insurance included specific questions on the premium exemption policy. The questions on the premium exemption policy were asked of all household members including older adults aged 70 years and older. The section on demographic characteristics of household members included the age, sex, marital status and level of education of household members.

### Sampling procedure

GLSS 7 was designed as a household probability survey that would provide regionally and nationally representative data on the various indicators measured in the survey. A two-stage stratified sampling design was employed in selecting respondents for the survey. The first stage of sampling involved the selection of 1000 randomly selected Enumeration Areas (EAs) sub-divided into 438 urban and 562 rural localities covering the entire country. These were selected to form the primary sampling units (PSUs) [19]. Selection of PSUs was stratified according to the 10 administrative regions with probability proportional to size. The sampling frame for the selection of PSUs was obtained from the 2010 Population and Housing Census. Following the selection of PSUs, a household listing exercise was conducted in each of the selected PSUs to obtain the secondary sampling units (SSUs). At the second stage of sampling, 15 households were selected from each secondary sampling unit. The sampling process resulted in the selection of a nationwide sample of 15,000 households [19]. Sampling weights that account for clustering in the sample design were incorporated into the selection of the sample for the survey.

### Study subjects

**Fig. 1:**
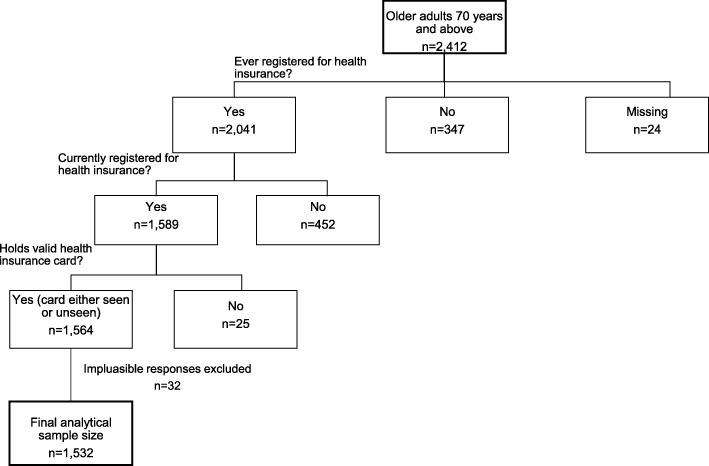
Diagrammatic presentation of application of eligibility criteria for selecting study sample

All individuals identified as household members in sampled households were listed in the household roster and questions on socio-demographic characteristics and health insurance were asked of each household member listed in the household roster. The subjects for this study include members of the household who were 70 years of age and older (*n* = 2412) and who had valid responses on questions regarding health insurance and socio-demographic characteristics (*n* = 2388). The age eligibility criterion of 70 years and above was applied in accordance with the age requirement for the old-age premium exemption policy under Ghana’s NHIS. In addition to the age criterion, the analysis was limited to older adults aged 70 years and above who had valid responses on being currently registered for health insurance at the time of the survey and had a valid health insurance card at the time of the survey (*n* = 1589). Upon further cleaning of the data, a final analytical sample of 1532 (unweighted) was realised after excluding responses for implausible categories for health insurance membership (exempt as under 18 years (*n* = 32)). Figure 1 shows how the inclusion criteria were applied to arrive at the final analytical sample size.

### Variables

**Fig. 2:**
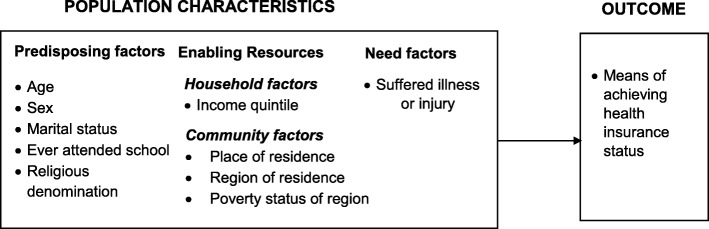
Conceptual framework of factors associated with the old-age premium exemption policy under Ghana’s NHIS*.* Adapted based on Andersen’s Behavioural Model of Health Service Use

This study adapted the Andersen’s Behavioural Model of Health Service Use [21] in conceptualising the factors associated with the old-age exemption policy (Fig. 2). The Andersen’s Behavioural Model has gone through several modifications since it was initially proposed in the 1960s [21] but is currently being applied to several areas of health services utilisation [22–26]. The original behaviour model distinguishes between three different dynamics (factors) under population characteristics that influence the actual use of health services. The first set of factors, so-called predisposing factors, covers demographic, social, and structural features, as well as health beliefs. The second factor grouping – enabling factors - encompasses everything that enables an individual person to actually use health services, for example money. Need represents the third factor which describes the need for services due to diseases or other disabilities. Those three factors do not only explain healthcare utilisation but can also guide the understanding of health insurance enrolment [18, 27]. In applying the model to the current study, old-age exemption is considered as the outcome variable that is influenced by a number of population characteristics including predisposing characteristics, enabling resources (both household and community factors) and need factors (Fig. 2).

#### Dependent variable

The outcome variable for this study is captured as “means of achieving NHIS membership”. This measure was assessed using the question that asked household members; *who paid the premium for your health insurance registration?* The response categories included paid myself, paid by friend/relative, paid by employers, paid by SSNIT and exempt as aged. The “exempt as aged” category, also represented as “exempted” or “exempt” was the main category of interest that was used in assessing the old-age exemption policy. For the purposes of this study, exempt as aged was categorised as exempt, while paid myself, paid by friend/relative, paid by employers and paid by SSNIT were categorised as “self-financing/other”.

#### Independent variables

The independent variables for this study include individual socio-demographic characteristics of the older adults which are conceptualised as population characteristics grouped under predisposing, enabling resources and need factors. The individual predisposing and enabling factors include age, sex, marital status, religious affiliation, level of education, and income quintiles of the household the older adults belong to. Community enabling factors include, place and region of residence as well as the poverty status of the region of residence. The need factor was assessed using a measure of health status of the older adult in the last 2 weeks preceding the survey; specifically, whether or not the older adult suffered any illness or injury in the last 2 weeks preceding the survey.

### Methods of analysis

The socio-demographic characteristics of the study subjects were described using descriptive statistical tools including means and percentages. Bivariate associations between the dependent variable and the independent variables were examined using chi-square analysis. The factors associated with old-age exemption were further investigated using binary logistic regression analysis. The analyses accounted for clustering in the sampling design by applying sampling weights and generating robust standard errors. Statistical analyses were performed in Stata version 14.

## Results

### Socio-demographic characteristics of study sample

The older adults in this study were about 78 years old on average with about two-thirds (64.47%) being 70 to 79 years old. About 6 in 10 were females and about another 6 in 10 were not in union at the time of the survey (Table 1). Christians were in the majority constituting nearly 70% of the total sample. A little over one-third (38.1%) of the older adults have ever attended school. Regarding their health status, about three-quarters of the older adults reported that they did not experience illness or injury in the last 2 weeks preceding the survey but a quarter of them experienced illness or injury in the same period. In terms of wealth status, a little more than one-fifth (22.7%) of the older adults belonged to households in the richest income quintile. And in terms of place of residence, there was an almost equal distribution of older adults in urban and rural areas. Respondents from the Ashanti region constituted the highest proportion of the entire study sample and nearly 8 out of 10 of the older adults were residents of regions classified as non-poor (Table 1).

**Table 1:** Sociodemographic characteristics and old-age premium exemption for NHIS

Variable	Mean ± SD/unweighted N (weighted %)
**Age (Mean ± SD)**	77.67 ± 6.71
**Age Group**	
70–79	981 (64.47)
80–89	459 (29.13)
90–99	92 (6.40)
**Sex**	
Male	549 (34.77)
Female	983 (65.23)
**Marital status**	
In union	614 (36.75)
Not in union	918 (63.25)
**Religious Denomination**	
No religion/Traditionalist	271 (10.93)
Christian	943 (69.26)
Muslim	318 (19.82)
**Ever attended school**	
Yes	450 (38.12)
No	1082 (61.88)
**Health status**	
No illness or injury	1125 (74.61)
Experienced illness/injury	407 (25.39)
**Income quintiles**	
1 (Poorest)	545 (19.66)
2	313 (19.38)
3	257 (18.36)
4	203 (19.92)
5 (Richest)	214 (22.68)
**Place of residence**	
Urban	517 (49.32)
Rural	1015 (50.68)
**Region of residence**	
Western	118 (8.11)
Central	97 (12.76)
Volta	199 (12.10)
Eastern	139 (14.67)
Ashanti	110 (16.74)
Brong Ahafo	122 (9.99)
Northern	135 (7.67)
Upper East	281 (7.21)
Upper West	265 (4.69)
Greater Accra	66 (6.08)
**Poverty status of region**	
Very Poor	300 (8.48)
Poor	303 (14.91)
Non-poor	929 (76.61)
**Means of achieving NHIS membership**	
Exempt	658 (42.84)
Self-financing/Other	874 (57.16)
**Total % (unweighted N)**	1532 (100.0)

*SD* Standard Deviation, *N* Number

### NHIS membership and payment of health insurance premiums

The results show only about 4 out of 10 older adults are taking up the old-age premium exemption and benefiting from the policy for NHIS membership (Table 2). More than half of the older adults still paid premiums for their NHIS membership despite the exemption. Specifically, a little more than one-third of the older adults indicated that they paid premiums for their NHIS membership themselves. Additionally, about one-fifth of the older adults reported that their NHIS premiums were paid by relatives or friends. For a small proportion of the older adults, their NHIS premiums were paid by others including employers or the social security and national insurance trust scheme (Table 2).

**Table 2:** Distribution of older persons by means of payment of premiums for NHIS membership

Payment of premiums for NHIS membership	Unweighted N (weighted %)
Exempt as aged	658 (42.84)
Paid myself (Self-financing)	529 (35.03)
Paid by a relative/friend	288 (18.97)
Paid by others^a^	57 (3.16)
**Total**	**1532 (100.00)**

^a^ Includes SSNIT, employers and other persons

The results further indicate that realisation of exemption was more common among older adults who were 90 to 99 years old and a higher proportion of females compared to males reported being exempted from paying premiums for their NHIS membership (Table 3). Regarding marital status, a higher proportion of those who were not in union at the time of the survey reported being exempt, and among the various religious groups, reported exemption was least common among Muslims. Also, a higher proportion of older adults who have had no formal education reported being beneficiaries of the exemption policy compared to their counterparts who have had some formal education. And in terms of health status, there was about a seven percentage point difference in the proportion that realised exemption among older adults who reported being ill or injured versus those who did not report any such condition. The distribution by wealth status does not show a discernible pattern but reported exemption was least common among older adults in the richest income quintile. The results further show realisation of exemption being slightly more common among older adults who live in urban areas but the regional distribution shows reported exemption being least common among older adults who live in the Northern region. Furthermore, while 39% of older adults in very poor regions acquired their health insurance membership through exemption, a slightly higher proportion of their counterparts in poor (44.22%) and non-poor (43.81%) regions reported being beneficiaries of exemption (Table 3).

**Table 3:** Socio-demographic factors associated with payment of NHIS premiums among older adults

Variable	Means of payment for NHIS premiums (%)	
	Exempt	Other
**Age**		
70–79	38.84	61.16
80–89	49.46	50.54
90–99+	54.35	45.65
**Sex**		
Male	39.89	60.11
Female	44.66	55.34
**Marital status**		
In union	40.07	59.93
Not in union	44.88	55.12
**Religious Denomination**		
No religion/Traditionalist	45.76	54.24
Christian	42.63	57.37
Muslim	41.51	58.49
**Ever attended school**		
Yes	38.44	61.56
No	44.82	55.18
**Health status**		
No illness or injury	41.16	58.84
Experienced illness/injury	47.91	52.09
**Income quintiles**		
1 (Poorest)	41.83	58.17
2	40.89	59.11
3	43.58	56.42
4	50.74	49.26
5 (Richest)	40.65	59.35
**Place of residence**		
Urban	45.26	54.74
Rural	41.77	58.23
**Region of residence**		
Western	48.31	51.69
Central	43.30	56.70
Volta	37.19	62.81
Eastern	56.83	43.17
Ashanti	39.09	60.91
Brong Ahafo	55.74	44.26
Northern	20.74	79.26
Upper East	51.96	48.04
Upper West	39.62	60.38
Greater Accra	24.24	75.76
**Poverty status of region**		
Very Poor	39.00	61.00
Poor	44.22	55.78
Non-poor	43.81	56.19
**Total %**	**42.84**	**57.16**

### Socio-demographic factors associated with old-age premium exemption

Table 4 shows the results of a binary logistic regression model that examines the population characteristics of older adults that influence their likelihood of realising and benefiting from the exemption from paying premiums under the NHIS. The results indicate that, as older persons (70 years and above) grow older, they are more likely to report being exempt from paying health insurance premiums for NHIS membership. But considering the sex of older adults, the chances of reporting exemption were not significantly different for women and men. Similarly, the odds of reporting exemption were not statistically different for older adults in union compared to those who were not in union. In terms of religious affiliation, there was no statistically significant difference in the odds of exemption for the Muslims or Christians when compared to traditionalists or those with no religious affiliation. Whether or not an older person has ever attended school was also not significantly associated with reporting exemption although those who have never attended school appeared to have a higher odds of reporting being exempted from paying premiums. But in terms of health status, older adults who experienced illness or injury in the last 2 weeks preceding the survey had a 45% higher chance of reporting exemption compared to their counterparts who did not experience any illness or injury. The results with regards to wealth status did not show a statistically significant association with reporting exemption but the results with regards to rural or urban residence shows that older adults residing in rural areas are significantly less likely to report exemption compared to older adults residing in urban areas. Furthermore, the results with regards to region of residence shows that older adults from all the various regions other than the Greater Accra region were significantly more likely to report exemption but statistical significance was not observed for the Northern region (Table 4). The poverty status of the region older adults reside in did not show a statistically significant influence on reported exemption although the pattern of results show a lower likelihood of realising exemption for poor and non-poor regions compared to very poor regions.

**Table 4:** Factors associated with old-age premium exemption among older persons (70 years and above) in Ghana

Variable ^[Reference Category]^	Odds ratio [95% CI]^***p*-value**^
**Age**	1.052 [1.029, 1.076]***
**Sex**^[Male]^	
Female	1.011 [0.702, 1.458]
**Marital status**^[In union]^	
Not in union	0.933 [0.663, 1.314]
**Religious Denomination**^[No religion/Traditionalist]^	
Christian	0.908 [0.599, 1.377]
Muslim	0.663 [0.407, 1.082]
**Ever attended school**^[Yes]^	
No	1.372 [0.944, 1.994]
**Health status**^[No illness/injury]^	
Experienced illness/injury	1.453 [1.053, 2.006]*
**Income quintiles**^[5(Richest)]^	
1 (Poorest)	0.874 [0.322, 2.373]
2	0.815 [0.486, 1.369]
3	1.308 [0.796, 2.147]
4	1.327 [0.802, 2.193]
**Place of residence**^[Urban]^	
Rural	0.581 [0.405, 0.833]**
**Region of residence**^[Greater Accra]^	
Western	4.603 [2.102, 10.079]***
Central	3.817 [1.627, 8.954]**
Volta	2.982 [1.379, 6.448]**
Eastern	7.344 [3.299, 16.350]***
Ashanti	3.196 [1.417, 7.207]**
Brong Ahafo	7.254 [3.325, 15.829]***
Northern	1.950 [0.806, 4.714]
Upper East	7.111 [3.210, 15.754]***
Upper West	3.715 [1.649, 8.370]**
**Poverty status of region**^[Very poor]^	
Poor	0.924 [0.567, 1.506]
Non-poor	0.665 [0.235, 1.879]

The regression model accounts for clustering and robust standard errors

*p < 0.05 **p < 0.01 ***p < 0.001

## Discussion

This study sought to examine the old-age premium exemption policy for health insurance membership under Ghana’s national health insurance programme. The findings indicate that less than half of the population of older adults aged 70 years and above who were members of the scheme at the time of the survey were actual beneficiaries of the old-age premium exemption policy. This implies that more than half of older adults who are eligible for exemption still pay premiums for their NHIS membership. Older adults who live in rural areas were observed to be less likely to benefit from the old-age premium exemption, suggesting an urban advantage in old-age premium exemption. This shows the old-age premium exemption policy is not working properly as it is not reaching the intended target beneficiaries. Several factors could account for these findings. One of the likely reasons for the low level of  exemption is the low level of awareness of the various exemption policies. In an assessment of the level of awareness of the various exemption policies, only about one third of users were aware of the old-age exemption policy [28]. Other studies have also found that older adults’ enrolment on the NHIS is generally low; particularly among the poorest [18]. These findings from other studies and the findings of the present study indicate the need to increase awareness about the old-age premium exemption.

Another plausible reason for the sub-optimal level of exemption among older adults may be due to mandatory requirements such as providing a proof of age [16]. Ghana introduced the registration of birth in 1912 yet the possession of birth certificates among poorer individuals is rare. Therefore, the implementation of the old age exemption is undermined [29].

Another barrier that may preclude older adults from realising the old-age premium exemption is the requirement of annual membership renewal. This presents a major barrier to older adults benefitting from the exemption policy because even though older adults are exempt from paying premiums, they are required to pay registration fees for the annual membership renewal [7, 13, 30] in order to have active/valid subscription on the NHIS. Even though the registration fee which is the equivalent of about $1 [30] is a relatively small cost, for older adults it is a substantial cost and a barrier to accessing the old-age exemption policy for NHIS membership. Kusi and Enemark [13] showed in their research that the registration fee imposes a burden specifically on very poor households. Moreover, difficulties in hearing, seeing, as well as moving, are common disabilities among older adults [31], which makes the renewal of the NHIS membership more difficult.

The results with regards to age show that for each additional increase in age, older adults are more likely to report being exempt from paying premiums. This means that older adults in the later years may have access to the much needed healthcare associated with ageing [7] through the old-age premium exemption policy. This finding is plausible because the need for healthcare may be higher at older ages and so older eligible adults will pursue the exemption policy as a more affordable means of access to healthcare.

The same however cannot be said when it comes to where an older person lives. The findings indicate that older adults who live in rural areas are at a disadvantage in terms of realising exemption. Other studies have also found that NHIS is higher among urban compared to rural residents for both young and older adults [27]. These findings suggest challenges in accessing healthcare for older adults living in rural areas. The healthcare needs of older adults in rural areas could be further compromised without the old-age premium exemption considering that rural areas in most African countries tend to be less developed compared to urban areas and urban areas also tend to have more facilities of higher quality which are more accessible compared to rural areas [7]. Other studies conducted in Ghana have found that NHIS enrolment among older people in rural areas is associated with the provision of health facilities and higher enrolment was observed in rural areas with health facilities [18]. As such, with the old-age exemption policy functioning poorly, coupled with limited access to health facilities and resources, older adults in rural areas are at risk of catastrophic out-of-pocket payments for healthcare. Similar to the urban rural differences observed, the results suggest regional variations in old-age premium exemption. The findings further highlight higher uptake of NHIS in the Eastern, Upper East and Brong Ahafo regions and lower uptake in the Greater Accra and Northern regions. There are several potential explanations for these regional differences. Dixon et al. [16] and van der Wielen et al. [32] found that adults living in the northern areas of Ghana have a greater likelihood of joining the NHIS. This could be due to earlier implementation of community-based insurance schemes in the regions in the north compared to the regions located in the south [16]. The three northern regions therefore had more time to institutionalise the concept of risk pooling and to build trust in a community-based insurance scheme.

## Conclusion

This study reveals that more than 5 out of 10 older adults, 70 years and older who are eligible for the old-age premium exemption policy under Ghana’s NHIS, are in reality not exempt. Nearly 60% of eligible older persons still self-finance their NHIS membership or have payments made for them by relatives, friends or employers. Furthermore, the policy is not reaching those who need it most, particularly those residing in rural areas. The old-age exemption policy is thus not achieving the intended goal of providing financial risk protection for older adults’, particularly rural dwellers. In order to achieve the aims of the old-age exemption policy, there is the need to reconsider how the policy is designed and implemented if the policy is to be effective. Deliberate efforts will be required to reach those older adults who report not being beneficiaries of the old-age premium exemption policy despite being eligible. Older adults in rural areas need to be targeted. Furthermore, requirements such as providing proof of age, annual renewal of membership and payment of registration fees need to be reviewed. Finally, there is the need for further research to investigate why some older adults still pay membership premiums despite being eligible for the old-age exemption policy. The question must be raised as to why the insurance scheme appears to be collecting premiums from a large number of older adults who are eligible for exemption. Beneficiaries are asked to provide proof of age on registration. While not all can do so, there should be a duty for registration offices to inform any prospective beneficiary of the exemption and instructions on how to provide proof of eligibility when registering.

## Data Availability

This study uses publicly available secondary data which can be accessed from the website of the Ghana Statistical Service on written request.

## Abbreviations

NHIS: National Health Insurance Scheme
OR: Odds ratios
SD: Standard Deviation
SSNIT: Social Security and National Insurance Trust
WHO: World Health Organisation
